# Correlation of Serum Prolactin Levels With Metabolic and Cardiovascular Risk in Greek Women With Polycystic Ovarian Syndrome

**DOI:** 10.7759/cureus.59430

**Published:** 2024-05-01

**Authors:** Konstantinos Kastrinakis, Sofoklis Stavros, Chrisi Christodoulaki, Eutychios Trakakis, Christos Tsagkaris, Sofia Kalantaridou, Georgios Mastorakos, Petros Drakakis, Periklis Panagopoulos

**Affiliations:** 1 Third Department of Obstetrics and Gynecology, Attikon University Hospital, National and Kapodistrian University of Athens, Athens, GRC; 2 Department of Obstetrics and Gynecology, Aghios Georgios General Hospital of Chania, Chania, GRC; 3 Public Health and Policy Working Group, European Student Think Tank, Amsterdam, NLD; 4 Unit of Endocrinology, Diabetes Mellitus, and Metabolism, Aretaieion Hospital, National and Kapodistrian University of Athens, Athens, GRC

**Keywords:** female reproductive system, metabolic disorders, cardiovascular disease, prolactine, polycystic ovarian syndrome

## Abstract

Background: Polycystic ovary syndrome (PCOS) is a common endocrine disorder among females. PCOS is associated with various metabolic and cardiovascular complications, including insulin resistance, dyslipidemia, and an increased risk of type 2 diabetes mellitus and cardiovascular disease. The role of serum prolactin (PRL) in the development of these complications in PCOS is not well understood.

Aim: This study aims to investigate the correlation between serum PRL levels and metabolic and cardiovascular risk factors in Greek women with PCOS.

Methods: The study utilized secondary outcomes from a prospectively collected patient database at the Third Department of Obstetrics and Gynecology, Medical School of the University of Athens. Data were collected from patients who visited the Gynecological Endocrinology - Pediatric and Adolescence Endocrinology Outpatient Clinic between January 2007 and December 2015. Measurements of various parameters, including PRL levels, BMI, waist circumference, hormone levels, lipid profiles, and insulin sensitivity, were obtained. Statistical analyses, including Mann-Whitney tests, chi-square tests, Spearman correlations, and multiple linear regression analyses, were conducted using SPSS software (IBM Corp., Armonk, NY, USA).

Results: The study included 247 women with PCOS, with a mean age of 24.7 years. Participants were divided into two groups based on the median PRL level. Women with higher PRL levels (>14.9) had lower BMI and waist circumference, higher levels of certain hormones and insulin sensitivity, and lower levels of fasting insulin, total cholesterol, and total lipids. Factors associated with lower PRL levels included being overweight/obese and smoking more than 10 cigarettes per day. Higher age, BMI, waist circumference, and certain hormone levels were associated with lower PRL levels.

Conclusion: The findings suggest a correlation between serum PRL levels and metabolic and cardiovascular risk factors in Greek women with PCOS. Further research is needed to elucidate the role of PRL in the pathophysiology of PCOS and to explore its potential as a diagnostic and therapeutic target.

## Introduction

Polycystic ovary syndrome (PCOS) is a common endocrine disorder that affects between 3-10% of women worldwide. In the WHO Europe region, PCOS is estimated to affect 5-15% of women, with the highest prevalence reported in southeastern Europe. In Greece, the prevalence of PCOS is estimated to be around 6-9%. PCOS is characterized by a combination of clinical, laboratory and radiological features, including menstrual irregularities, hyperandrogenism, and polycystic ovarian morphology on ultrasound. In addition, PCOS is associated with metabolic and cardiovascular morbidity, including insulin resistance, dyslipidemia, and an increased risk of type 2 diabetes mellitus, hypertension, and cardiovascular disease [1].

Some of the most common metabolic disorders associated with PCOS include insulin resistance, impaired glucose tolerance, dyslipidemia, and type 2 diabetes mellitus. Insulin resistance is reported in 50-70% of women with PCOS, and up to 10% of women with PCOS may develop type 2 diabetes by the age of 40. Dyslipidemia, characterized by elevated triglycerides and low levels of high-density lipoprotein (HDL) cholesterol, is present in 70-80% of women with PCOS [2].

PCOS is also associated with an increased risk of cardiovascular disease (CVD), including hypertension, left ventricular hypertrophy, and coronary artery disease. The prevalence of hypertension in women with PCOS is estimated to be 30-40%, which is higher than the general population. The prevalence of left ventricular hypertrophy is three to four times higher in women with PCOS compared to women without the condition [3].

The mechanistic underpinnings of the involvement of PCOS in cardiometabolic disorders are not entirely understood. A growing body of evidence tends to investigate serum prolactin (PRL) as a potential mediator or biomarker of the cardiovascular and metabolic deregulation in women with PCOS. PRL is a hormone produced by the pituitary gland that is physiologically associated with the regulation of reproductive function and lactation. Elevated PRL levels have been reported in a subset of women with PCOS. On this basis, it has been hypothesized that PRL may be implicated in the pathogenesis of metabolic and cardiovascular complications in women suffering from PCOS. However, the association between serum PRL and cardiometabolic risk in this demographic remains opaque, urging for further research to elucidate the role of PRL in PCOS [4,5].

The aim of the present study is to investigate the correlation between serum PRL levels and metabolic and cardiovascular risk factors in Greek women with PCOS. Understanding the role of PRL in the development of metabolic and cardiovascular complications in women with PCOS may provide insights into the pathophysiology of this complex disorder and may ultimately lead to the development of more effective diagnostic and therapeutic approaches.

## Materials and methods

Patient selection

The study was conducted in the Third Department of Obstetrics and Gynecology of the Medical School of the University of Athens and has been approved by the Institution Ethics Committee (Μ/Γ,ΕΒΔ313/08-05-2023). It utilized secondary outcomes obtained from a patient database that was prospectively collected. The selection criteria have been previously described in detail [5]. In principle, patients who visited the Gynecological Endocrinology - Pediatric and Adolescence Endocrinology Outpatient Clinic at our hospital between January 2007 and December 2015 (n=367) were screened. After baseline assessment, 321 of the aforementioned patients, that met the Rotterdam (ESHRE/ASRM - sponsored) PCOS criteria were included in the study. Patients were excluded based on a prior history of underlying conditions that could cause hyperandrogenemia or ovarian dysfunction, namely congenital adrenal hyperplasia, Cushing syndrome, malignancies, thyroid disorders and hyperprolactinaemia. Underage (18 years old) as well as adult patients who refused to participate in the present study were also excluded.

Clinical, laboratory and imaging data collection

Information regarding the medical history, including characteristics of the menstrual cycle, accompanying diseases, and daily activities such as alcohol consumption and cigarette smoking, was recorded. Obesity was defined based on the calculation of the body mass index (BMI) using a threshold of 30, as suggested by the World Health Organization (WHO). Interviews and clinical measurements to assess the aforementioned parameters were performed during a series of dedicated visits to our outpatient clinic.

Laboratory tests were conducted in the Biopathology laboratory of the Attikon University Hospital. During the early follicular phase (i.e., between days three and six of the menstrual cycle) morning fasting blood samples were collected. Luteinizing hormone (LH), follicular stimulating hormone (FSH), total testosterone (T), free testosterone (fT), 17-b estradiol (E2), 17OH-progesterone (17OH-P), PRL, sex hormone binding globulin (SHBG), Δ4-androstenedione, cortisol (E), dehydroepiandrosterone sulphate (DHEAS), cholesterol (Chol), HDL, low-density lipoproteins (LDL), triglycerides (TG), glucose (Glu) and insulin were isolated and measured by means of centrifugation.

To assess patients for impaired glucose tolerance (IGT) and insulin resistance (IR) an oral glucose tolerance test (OGTT) with 75gr glucose following an eight- to 12-hour fasting with insulin and glucose levels being tested at 0 minutes, 60 minutes and 120 minutes after glucose uptake. The diagnosis of IR was based on the presence of one of the two following criteria: serum insulin levels at 120 minutes > 100 μIU/ml or serum insulin levels at 120 minutes 10-fold higher than at 0 minutes. Insulin resistance was further assessed by means of the Homeostasis Model Assessment for Insulin Resistance (HOMA-IR), the Quantitative Insulin Sensitivity Check Index (QUICKI) and Matsuda index. The relevant calculation formulas have been presented in a previous study from our department.

Additionally, ovarian morphology was assessed by means of a transvaginal ultrasound examination between the sixth and the eighth day of the menstrual cycle. The examination was performed by consultant obstetrician-gynaecologists who served in our department at the time of the study with a General Electrics Voluson 730 ultrasound machine (General Electrics, Boston, MA, USA).

The participants were subsequently divided into two groups based on the median PRL level. Their characteristics were compared with regard to the BMI, waist circumference, levels of OHP17, SHBG, blood cortisol, fasting insulin, insulin at 120 minutes following glucose intake, total cholesterol, total lipids, apolipoprotein beta (APO-b), thyroid-stimulating hormone (TSH), and the Matsuda index.

## Results

Our sample consisted of 247 women with a mean age of 24.7 years (SD=5.5 years). Participants’ clinical and biochemical data are presented in Table 1. Most women (56.2%) had a normal BMI and 27.7% were smokers. Mean PRL was 17.4 (SD=10) and its median value was 14.9.

**Table 1:** Participants’ clinical and biochemical data FSH: follicular stimulating hormone, LH: luteinizing hormone, PRL: prolactin, E2: 17-b estradiol, 17OH-P: 17OH-progesterone, DHEAS: dehydroepiandrosterone sulphate, SHBG: sex hormone binding globulin, TSH: thyroid-stimulating hormone, HDL: high-density lipoproteins, LDL: low-density lipoproteins, TG: triglycerides, Lpa: lipoprotein (a), APO: apolipoprotein, HOMA-IR: Homeostasis Model Assessment for Insulin Resistance, QUICKI: Quantitative Insulin Sensitivity Check Index

	Mean (SD)
Age	24.7 (5.5)
ΒΜΙ	25.4 (6.4)
Waist circumference	89.5 (15.1)
Hip circumference	80 (28.8)
Waist/ hip ratio circumference	1.01 (0.37)
	N (%)
ΒΜΙ	
Underweight	13 (5.4)
Normal	136 (56.2)
Overweight	35 (14.5)
Obese	58 (24.0)
Smoking	65 (27.7)
If yes, daily number of cigarettes	
1-5	15 (23.1)
6-10	17 (26.2)
11-20	25 (38.5)
>20	8 (12.3)
	Median (IQR)
FSH (m/U/ml)	5.6 (4.4 ─ 6.7)
LH (m/U/ml)	5.5 (3.9 ─ 7.8)
PRL	14.9 (10.9 ─ 21.1)
E2 (pg/ml)	39.9 (31.2 ─ 52.3)
Total testosterone (ng/dl)	61.5 (43.5 ─ 77)
Free testosterone (pg/ml)	2 (1.45 ─ 2.74)
OHP17 (ng/ml)	1.2 (0.73 ─ 1.6)
DHEAS (μg/dl)	216 (142.6 ─ 316)
Δ4 androstenedione (ng/mg)	2.4 (1.8 ─ 3.3)
SHBG (nmol/L)	42 (29.5 ─ 61)
Blood cortisole (mg/dl)	19.4 (13.4 ─ 23.9)
T3 (nmol/L)	1.48 (1.2 ─ 1.97)
T4 (μg/dl)	7.8 (7 ─ 8.8)
TSH (mU/L)	1.98 (1.36 ─ 2.6)
Fasting glucose (mg/dl)	81 (73 ─ 88)
Glucose 120 min (mg/dl)	92 (75 ─ 112)
Fasting insulin (μ/U/ml)	10.5 (7 ─ 15.2)
Insulin 120 min (μ/U/ml)	52.4 (26.1 ─ 85)
Total cholesterol (mg/dl)	178 (158 ─ 198)
HDL (mg/dl)	56 (46 ─ 66)
LDL (mg/dl)	106.3 (88 ─ 124.5)
TG (mg/dl)	72 (55 ─ 98)
Total lipids	477 (435.5 ─ 528.5)
Lpa	10.5 (6.5 ─ 40.9)
APO-a1 (mg/dl)	135 (121 ─ 150.5)
APO-b (mg/dl)	77 (60 ─ 90.9)
HIRSUTISM INDEX (LORENZO)	10 (6 ─ 12)
HOMA-IR score	1.91 (1.21 ─ 2.86)
QUICKI score	0.35 (0.33 ─ 0.37)
MATSUDA score	9.2 (5 ─ 13.6)

Patients with high PRL levels

When participants were grouped according to median PRL, it was found that the percentage of women with PRL >14.9 was significantly lower in overweight/obese women as well as in smokers who smoked more than 10 cigarettes per day (Table 2). A PRL level of 14.9 was defined as a cut-off value. Women with PRL >14.9 exhibited significantly lower BMI and waist circumference. Furthermore, women with PRL >14.9 had significantly greater OHP17, SHBG, blood cortisol, TSH and MATSUDA score. On the other hand, the same group exhibited significantly lower fasting insulin, insulin 120 min, total cholesterol, total lipids and APO-b.

**Table 2:** Participants’ clinical and biochemical data according to median prolactin level (14.9 mg/l). Mann–Whitney test and Chi square test were used to compare differences between the two levels of PRL. FSH: follicular stimulating hormone, LH: luteinizing hormone, PRL: prolactin, E2: 17-b estradiol, 17OH-P: 17OH-progesterone, DHEAS: dehydroepiandrosterone sulphate, SHBG: sex hormone binding globulin, TSH: thyroid-stimulating hormone, HDL: high-density lipoproteins, LDL: low-density lipoproteins, TG: triglycerides, Lpa: lipoprotein (a), APO: apolipoprotein, HOMA-IR: Homeostasis Model Assessment for Insulin Resistance, QUICKI: Quantitative Insulin Sensitivity Check Index

	PRL		
	≤14.9 N=124; 50.2%	>14.9 N=123; 49.8%	P
ΒΜΙ, N(%)			
Underweight/Normal	64 (52.0)	85 (71.4)	0.002
Overweight/Obese	59 (48.0)	34 (28.6)	
Smoking, N(%)			
No	84 (70.6)	86 (74.1)	0.543
Yes	35 (29.4)	30 (25.9)	
If yes, daily number of cigarettes, N(%)			
1-10	12 (34.3)	20 (66.7)	0.009
>10	23 (65.7)	10 (33.3)	
Age, mean (SD)	25.3 (5.7)	24.1 (5.2)	0.131
ΒΜΙ, mean (SD)	26.6 (6.8)	24.1 (5.7)	0.004
Waist circumference, mean (SD)	93.7 (15.8)	85.4 (13.2)	<0.001
Hip circumference, mean (SD)	80.4 (28.5)	79.6 (29.2)	0.386
Waist/hip ratio circumference, mean (SD)	1.04 (0.37)	0.99 (0.37)	0.355
FSH (m/U/ml), median (IQR)	5.5 (4.4 ─ 6.5)	5.7 (4.4 ─ 6.9)	0.209
LH (m/U/ml), median (IQR)	5.5 (3.4 ─ 7.8)	5.6 (4.2 ─ 7.8)	0.145
E2 (pg/ml), median (IQR)	37.5 (31 ─ 50)	40 (32 ─ 53.1)	0.508
Total testosterone (ng/dl), median (IQR)	60.5 (41 ─ 74)	62 (49.5 ─ 78)	0.278
Free testosterone (pg/ml), median (IQR)	2 (1.3 ─ 2.7)	2.1 (1.6 ─ 2.8)	0.245
OHP17 (ng/ml), median (IQR)	0.96 (0.64 ─ 1.4)	1.35 (0.9 ─ 2.1)	<0.001
DHEAS (μg/dl), median (IQR)	211.6 (154.4 ─ 315.9)	227 (129.6 ─ 316)	0.707
Δ4 androstenedione (ng/mg), median (IQR)	2.32 (1.74 ─ 3.2)	2.45 (1.99 ─ 3.5)	0.264
SHBG (nmol/L), median (IQR)	36.5 (27 ─ 59)	48 (34.8 ─ 61)	0.035
Blood cortisol (mg/dl), median (IQR)	15.7 (12 ─ 20)	21.9 (15.5 ─ 25.1)	<0.001
T3 (nmol/L), median (IQR)	1.64 (1.22 ─ 2)	1.31 (1.1 ─ 1.86)	0.043
T4 (μg/dl), median (IQR)	8.2 (7 ─ 8.9)	7.6 (6.7 ─ 8.3)	0.075
TSH (mU/L), median (IQR)	1.81 (1.19 ─ 2.44)	2.04 (1.6 ─ 2.69)	0.026
Fasting glucose (mg/dl), median (IQR)	82 (74 ─ 88)	81 (73 ─ 88)	0.799
Glucose 120 min (mg/dl), median (IQR)	97 (74 ─ 116)	88 (76 ─ 103)	0.131
Fasting insulin (μ/U/ml), median (IQR)	11.6 (7.7 ─ 16.7)	9.4 (6.9 ─ 14)	0.033
Insulin 120 min (μ/U/ml), median (IQR)	64 (32 ─ 116)	37.6 (24.8 ─ 66.8)	0.001
Total cholesterol (mg/dl), median (IQR)	180 (161 ─ 209)	172 (153.5 ─ 192)	0.018
HDL (mg/dl), median (IQR)	56.5 (46 ─ 65)	56 (46 ─ 66.5)	0.494
LDL (mg/dl), median (IQR)	111 (90 ─ 130)	101 (84 ─ 120)	0.171
TG (mg/dl), median (IQR)	81 (52 ─ 110)	70 (57 ─ 89)	0.463
Total lipids, median (IQR)	495.5 (450 ─ 584)	455.5 (429 ─ 494)	0.026
Lpa, median (IQR)	7.4 (5.9 ─ 42.5)	11 (8 ─ 40.9)	0.224
APO-a1 (mg/dl), median (IQR)	135 (126.5 ─ 148.6)	133 (118.9 ─ 153)	0.916
APO-b (mg/dl), median (IQR)	82.5 (61 ─ 98)	75 (59 ─ 85)	0.034
HIRSUTISM INDEX (LORENZO), median (IQR)	10 (6 ─ 12)	10 (6 ─ 12)	0.906
HOMA-IR score, median (IQR)	2.1 (1.3 ─ 2.9)	1.8 (1.2 ─ 2.8)	0.183
QUICKI score, median (IQR)	0.34 (0.33 ─ 0.37)	0.35 (0.33 ─ 0.37)	0.183
MATSUDA score, median (IQR)	7.1 (4.6 ─ 11.6)	9.9 (6 ─ 15.9)	0.035

Patients with low PRL levels

PRL was significantly lower in overweight/obese women and in smokers who smoked more than 10 cigarettes per day (Table 3). Greater age, BMI and waist circumference were significantly associated with lower PRL. Moreover, greater T3, T4, insulin 120 min, total cholesterol and total lipids were significantly associated with lower PRL. On the contrary, higher OHP17, Δ4 androstenedione, SHBG, blood cortisol, TSH and MATSUDA score were significantly associated with higher PRL. After adjusting for smoking, age and BMI, OHP17, T4, TSH and total lipids remained significantly correlated with PRL, in a similar to the univariate way (Table 4).

**Table 3:** Bivariate associations between prolactin and participants’ clinical and biochemical data Mann–Whitney test was used to compare differences in PRL between two groups and Spearman’s rho coefficients for the correlation of quantitative variables with PRL. FSH: follicular stimulating hormone, LH: luteinizing hormone, PRL: prolactin, E2: 17-b estradiol, 17OH-P: 17OH-progesterone, DHEAS: dehydroepiandrosterone sulphate, SHBG: sex hormone binding globulin, TSH: thyroid-stimulating hormone, HDL: high-density lipoproteins, LDL: low-density lipoproteins, TG: triglycerides, Lpa: lipoprotein (a), APO: apolipoprotein, HOMA-IR: Homeostasis Model Assessment for Insulin Resistance, QUICKI: Quantitative Insulin Sensitivity Check Index

	Median PRL (IQR)	P^+^
ΒΜΙ		
Underweight/Normal	17.0 (11.7 ─ 22.5)	0.002
Overweight/Obese	12.4 (9.5 ─ 19.0)	
Smoking		
No	15.1 (11.0 ─ 22.0)	0.211
Yes	13.7 (10.1 ─ 19.4)	
If yes, daily number of cigarettes		
1-10	17.45 (10.5 ─ 22.0)	0.050
>10	12.5 (9.5 ─ 16.3)	
	rho	
Age	-0.14	0.024
ΒΜΙ	-0.17	0.008
Waist circumference	-0.21	0.002
Hip circumference	-0.06	0.406
Waist/hip ratio circumference	-0.08	0.429
FSH (m/U/ml)	0.03	0.597
LH (m/U/ml)	0.08	0.241
E2 (pg/ml)	0.08	0.233
Total testosterone (ng/dl)	0.07	0.311
Free testosterone (pg/ml)	0.08	0.260
OHP17 (ng/ml)	0.26	<0.001
DHEAS (μg/dl)	-0.03	0.661
Δ4 androstenedione (ng/mg)	0.13	0.048
SHBG (nmol/L)	0.15	0.048
Blood cortisol (mg/dl)	0.33	<0.001
T3 (nmol/L)	-0.17	0.028
T4 (μg/dl)	-0.16	0.044
TSH (mU/L)	0.17	0.011
Fasting glucose (mg/dl)	-0.05	0.414
Glucose 120 min (mg/dl)	-0.07	0.304
Fasting insulin (μ/U/ml)	-0.11	0.105
Insulin 120 min (μ/U/ml)	-0.20	0.006
Total cholesterol (mg/dl)	-0.18	0.014
HDL (mg/dl)	0.08	0.307
LDL (mg/dl)	-0.08	0.306
TG (mg/dl)	0.01	0.938
Total lipids	-0.23	0.040
Lpa	0.15	0.384
APO-a1 (mg/dl)	-0.07	0.438
APO-b (mg/dl)	-0.11	0.269
HIRSUTISM INDEX (LORENZO)	0.02	0.748
HOMA-IR score	-0.10	0.187
QUICKI score	0.10	0.188
MATSUDA score	0.16	0.046

**Table 4:** Regression analyses on the effect of prolactin on hormonal and metabolic outcomes after adjusting for smoking, age and BMI +regression coefficient ++Standard Error PRL: prolactin, SHBG: sex hormone binding globulin, TSH: thyroid-stimulating hormone

Dependent variables	Independent variables	β+	SE++	P
OHP17 (ng/ml)				
	PRL	0.019	0.005	0.001
	Age	-0.019	0.011	0.079
	ΒΜΙ	-0.001	0.009	0.933
	Smoking (yes vs no)	0.394	0.129	0.003
Δ4 androstenedione (ng/mg)				
	PRL	0.014	0.009	0.120
	Age	-0.019	0.017	0.289
	ΒΜΙ	0.000	0.015	0.999
	Smoking (yes vs no)	0.808	0.207	<0.001
SHBG (nmol/L)				
	PRL	0.042	0.165	0.798
	Age	0.944	0.346	0.007
	ΒΜΙ	-1.660	0.296	<0.001
	Smoking (yes vs no)	-1.233	4.018	0.759
Blood cortisol (mg/dl)				
	PRL	0.190	0.263	0.472
	Age	0.067	0.537	0.900
	ΒΜΙ	-0.048	0.468	0.918
	Smoking (yes vs no)	4.529	6.544	0.490
T3 (nmol/L)				
	PRL	-0.007	0.004	0.106
	Age	-0.003	0.007	0.640
	ΒΜΙ	0.013	0.006	0.028
	Smoking (yes vs no)	-0.068	0.088	0.445
T4 (μg/dl)				
	PRL	-0.029	0.015	0.050
	Age	-0.011	0.026	0.682
	ΒΜΙ	0.044	0.022	0.052
	Smoking (yes vs no)	0.457	0.323	0.159
TSH (mU/L)				
	PRL	0.022	0.011	0.041
	Age	-0.009	0.022	0.680
	ΒΜΙ	-0.007	0.018	0.705
	Smoking (yes vs no)	-0.151	0.255	0.555
Insulin 120 min (μ/U/ml)				
	PRL	-0.222	0.550	0.687
	Age	-3.299	1.000	0.001
	ΒΜΙ	6.046	0.802	<0.001
	Smoking (yes vs no)	-8.020	11.336	0.480
Total cholesterol (mg/dl)				
	PRL	-0.316	0.221	0.155
	Age	1.391	0.422	0.001
	ΒΜΙ	1.008	0.348	0.004
	Smoking (yes vs no)	-3.803	5.100	0.457
Total lipids				
	PRL	-2.892	1.297	0.029
	Age	5.065	2.323	0.033
	ΒΜΙ	2.947	1.996	0.144
	Smoking (yes vs no)	39.296	29.225	0.183
MATSUDA score				
	PRL	0.022	0.057	0.694
	Age	0.093	0.096	0.336
	ΒΜΙ	-0.527	0.085	<0.001
	Smoking (yes vs no)	-0.174	1.124	0.877

## Discussion

The present study investigated the correlation between serum prolactin levels and metabolic and cardiovascular risk factors in Greek women with PCOS. Our results showed that overweight/obese women and smokers who smoked more than 10 cigarettes per day had lower levels of prolactin, while higher levels of prolactin were associated with better metabolic profiles and lower cardiovascular risk. These findings are consistent with previous studies that have demonstrated the association between low prolactin levels and increased risk of cardiovascular disease and metabolic disorders, such as insulin resistance and dyslipidemia [4,5].

The observed association between low prolactin levels and increased cardiovascular risk may be explained by the known physiological effects of prolactin on the cardiovascular system, including its anti-inflammatory and anti-atherosclerotic properties [6]. Furthermore, low prolactin levels have been associated with impaired glucose metabolism and insulin resistance, both of which are important risk factors for the development of type 2 diabetes [7].

Our findings bring about previous studies that have shown an association between smoking and increased prolactin levels in women [8]. Smoking is a known risk factor for cardiovascular disease and metabolic disorders, and our results suggest that this may be partially mediated by its effects on prolactin levels.

Interestingly, we found that higher prolactin levels were associated with lower BMI and waist circumference, as well as higher levels of SHBG, OHP17, and cortisone. These results suggest that prolactin may play a role in regulating body weight and metabolic function, as well as modulating the activity of the hypothalamic-pituitary-adrenal axis. These findings are in principle consistent with previous studies that have demonstrated the role of prolactin in regulating insulin sensitivity and glucose metabolism [9]. Although evidence related to the metabolic implications of hyper- or hypoprolactinemia in this demographic derives primarily from clinical studies, Mastnak et al. (2023) have summarised evidence concerning its mechanistic underpinnings. Physiologically, PRL supports pancreatic islet growth, prevents ß-cell apoptosis, and stimulates insulin secretion. These effects are induced by a cascade of interactions that involve upregulation of osteoprotegerin synthesis, which in turns promotes ß-cell replication and inhibition of the respective apoptotic pathways. In isolated human pancreatic islets, prolactin inhibits apoptosis pathways and enhances glucose sensitivity by increasing glucose transporter 2 and glucokinase expression. Additionally, prolactin may be implicated in lipid storage within the liver by inhibiting fatty acid transketolase and stearoyl-CoA desaturase 1 expression. This justifies the optimal metabolic regulation among individuals with increased levels of prolactin and is also compatible with overweight and obesity documented among patients with hypoprolactinemia. Nevertheless, it cannot be safely assumed that excessive levels of PRL are beneficial metabolically. Although this has not been studied to our knowledge, a down-regulating effect of hyperprolactinemia could potentially lead to unfavorable metabolic regulation comparable to the one induced in the context of hypoprolactinemia [10].

The clinical significance of this study lies in its potential to inform clinical practice and management of women with PCOS. The findings suggest that serum PRL levels may be a useful biomarker for identifying women with PCOS who are at increased risk for metabolic and cardiovascular complications. This information can be used to guide early interventions and prevent the development of these complications. Given that in our practice, this disorder is primarily diagnosed and treated by obstetricians and gynecologists the involvement of specialists in metabolism, either internal medicine physicians or endocrinologists, would be essential to tackle the implications of metabolic disorders, prevent and early diagnose and address diseases in the context of metabolic syndrome. This can be achieved using referral to the respective specialists or interdisciplinary consultations involving both domains. Consequent follow-up either within specialized outpatient clinics or in collaboration with primary healthcare providers in the community would be pivotal for the continuum of treatment. Capitalizing on the need to provide holistic and patient-centered care, treatment in the community should harness capacities found within multidisciplinary primary care teams involving specialists in nutrition, physiotherapists and psychologists. Their involvement holds a major potential to finetune the finetuning of eating habits and physical activity in the daily lives of affected patients. At a broader level, social prescription of exercise could further support the control and prevention of metabolic dysregulation sequelae including overweight and obesity. The latter is certainly pending to the regulation of social prescription by the respective healthcare authorities. Evaluating the outcomes of the aforementioned interventions and integrating them into clinical guidelines after careful deliberation is important. Additionally to these, involving patients' associations in efforts to advocate for continuous and multidisciplinary care should be considered. 

Additionally, these findings add up to a consequent effort to create evidence at the local level, particularly in Athens, Greece, at a tertiary center that attends to the health needs of the most densely populated region of the country while serving as a referral center for primary and secondary healthcare facilities within the first and second regional healthcare administration districts of the country. This rationale is aligned with the WHO's call to enhance efforts to provide health for all taking into consideration local and regional health determinants, risk factors, lifestyles and healthcare services provision. This framework has already reported on the interrelation between PCOS and metabolic profiles [11-16]. 

One limitation of our study is its cross-sectional design, which does not allow us to establish a causal relationship between prolactin levels and cardiovascular and metabolic risk factors. Additionally, our study was conducted in a relatively small sample of Greek women with PCOS, being monitored in a single center, which may limit the generalizability of our findings to other regions and populations. Future prospective studies with larger sample sizes are needed to confirm the association between prolactin levels and cardiovascular and metabolic risk in women with PCOS.

## Conclusions

In conclusion, our study provides further evidence for the association between low prolactin levels and increased cardiovascular and metabolic risk in women with PCOS. Our findings suggest that prolactin may play a role in regulating metabolic and cardiovascular health in women with PCOS, and may have implications for the development of targeted interventions to improve the health outcomes of this patient population.
